# Construction Notes

**Published:** 1893-08-26

**Authors:** 


### CONSTRUCTION NOTES.

### Dunfermline Cottage Hospital.

The site selected for this building measures 280 ft. by 150 ft. The entrance is on the north part, thus leaving all the south part free for the wards. Accommodation has been formed in the front basement for a large kitchen and offices, including coal and heating apparatus. These have been made sufficiently large to meet the requirements of a further extension of the wards. A lift is provided to the floor above for dinner service. The main floor enters from the north front, and leads into a spacious entrance hall with staircase to the floor above and below, and with passages right and left to the men's and women's wards respectively.

These are planned each for six beds, and with the necessary lavatory and bath accommodation entering off each. One hundred superficial feet of floor space is allowed for each patient, and a cubic space of 2,000 ft., whilst fresh air inlets and induced extraction of the foul air of the wards gives a constant change of atmosphere. On the principal floor there are rooms for Doctor and Matron, and an operating-room on the floor above, also two small observation wards of two beds each, for men and women respectively, with bath and lavatory accommodation, and nurses and servants' rooms. The building is designed with due regard to economy, with an effective treatment of the roofs, without any elaborate architectural details. It is intended to build the necessary laundry offices at the north-east corner of the ground. The estimated cost is £2,700. The architects are Messrs. Sydney Mitchell and Wilson, of Edinburgh, and the mason work has been effected by Mr. Stewart, of Dunfermline.

**New Small-Pox Hospital, Birmingham.**

Plans for the erection of this building have been accepted from Mr. W. H. Ward, the architect of the Parish Infirmary. It is proposed to erect two pavilions, each to accommodate twenty-six patients. The buildings, which lie well back from the road, will be of a substantial character and equipped with everything adapted to promote the recovery of the inmates of the institution. The site chosen is distant three and a-half miles from the centre of the town.

**New Hospital and Dispensary, Scarborough.**

For a long time it has been felt by the Board that the existing premises of the dispensary were insufficient in size, and a scheme was set on foot for the erection of a larger institution. Plans for this were accepted from Messrs. Hall and Tugwell. The design shows a building of red brick, with local stone dressings and bay windows. On the ground floor are the dining hall, board room, matron's room, and apartments specially set apart for the house surgeon at the east end of the block; whilst the dispensary and consulting and waiting rooms are at the west end. The kitchens are placed at the rear of the matron's and surgeon's rooms, being cut off by a corridor. They are all that can be desired, and, with the rooms already referred to, form a well-arranged and easily-managed administrative block. The staircase is of stone, and rises from the central hall opposite the main entrance to the various floors, and arrangements and space are also left to add the lift provided for by the architects, the construction of which, it is hoped, will shortly be completed. The whole of the wards on the first floor face the south, and are designed especially for women and children. At the east end is the medical ward of six beds, with bath-rooms and lavatories adjoining, also a surgical ward of three beds, and another room of two beds for special purposes. The south end of the building comprises the children's ward of six beds, with baths and other conveniences adjoining, and in close proximity are the nurses' rooms and matron's bed-room. In front of the children's room is provided a balcony for patients. The second floor is given up to the use of men, and faces due south. At the east end of the block is the medical ward, for six beds, baths, lavatories, &c., in connection. Rooms with separate baths for the medical staff are here also. The surgical ward at the south end contains eight beds, and baths, operating-room, and separate bedrooms. The mortuary, post-mortem, soiled linen, and disinfectant rooms are placed at the rear of the building, and have a separate entrance. A large waiting-room has been provided for outdoor patients, for which a side door is constructed. The whole of this part of the building is isolated from the hospital proper. The building is heated with open fireplaces, the heating chamber being in the basement, with a steam disinfecting injector attached for disinfection of linen. The drainage has been carried out with all latest improvements, provision being made for a continual current of fresh air to pass through all the drainage pipes connected with the premises. The contract for the building was let to Mr. John Barry, the steam pipes and heating arrangements being carried out by Messrs. F. Ashwell and Co., of London.

